# Ship Fever, So-called: Its History, Nature, and Best Treatment

**Published:** 1850-07

**Authors:** 


### BIBLIOGRAPHICAL NOTICES.

*Ship Fever, so called: its History, Nature, and best Treatment. The Fiske Fund Prize Dissertation for* 1849. By Henry Grafton Clark, M. D., Member of the Boston Society for Medical Improvement. 8vo. pp. 48. *With a plate.* Boston, 1850.

This essay is the result of observations made by its author, as visiting physician of the Deer Island Hospital. This establishment being designed for the reception of sick emigrants, afforded an excellent field for the study of this disease, the prevailing one among them. It is generated, according to our author, not by the scarcity or bad quality of their food, but by the bad atmosphere resulting from overcrowding and filthy habits. He thus describes a scene he witnessed on one of the emigrant vessels. "There were fifty or sixty sick persons in various stages of fever, some with the dysentery, which sometimes follows as a secondary attack, lying in their berths indiscriminately together, adults and children, without the least care or attention to cleanliness, and as, we were assured, they had done during the continuance of the whole voyage! In another berth were the bodies of two children, who had died within an hour or two of our arrival; and close by them, and almost in contact, the feeble mother at whose dry and withered breast dragged a miserable and squalid infant. Those who were able, were staggering about with haggard countenances, whose starting eyes and hollow cheeks showed how narrow an escape they had themselves had from the lonely grave in mid-ocean, which their associates and their relations had long since found."

Dr. Clark alludes to many facts to prove the contagious nature of this fever, and states that in the hospital under his care all the nurses had been attacked, the physician had died, and the only remaining assistant physician and apothecary were sick. He believes also that it may be propagated by chests of clothing. Very praiseworthy measures were adopted to ensure complete ventilation and cleanliness, after the hospital was placed under the care of Dr. Clark. The air of the hospital was constantly renewed, the fever patients in the acute stage were washed every morning, deodorizing agents employed, and all unnecessary articles of clothing removed from the wards. Every patient was washed before admission into the house, and their clothing entirely changed. The success of these measures was attested by the diminished mortality, and by the subsequent immunity from contagion enjoyed by the officers, attendants, and visitors to the hospital. Our author believes that a first attack preserves, as a general rule, from a second one, an opinion which he grounds upon a personal familiarity with nearly two thousand cases. He was guided in the selection of his nurses by a knowledge of the fact. The ordinary limit of the disease, he says, is fourteen or fifteen days, but two-thirds of all the deaths occur at a later period. This mortality is caused by the relapses, or, as they are termed by our author, secondary diseases, which he classes under two principal forms, which, though generally distinct, sometimes run into each other.

"1. General dropsy, which is often accompanied by swelling and sloughing, or suppuration of the parotid and other glands, and occasionally by suffocative œdema of the glottis.

"2. A diarrhœa or dysentery, which is usually dependant upon inflammation and ulceration of the ileum and cœcum and is frequently fatal."

Three cases are reported in full, to illustrate the history of the latter of these secondary affections; and in connexion with this description, Dr. Clark remarks that it is probable "that some of the cases which have been set down as typhoid, (merely on account of the presence of this symptom during life, unverified by a post-mortem examination,) were really the sequelæ of typhus." Also, "It must then be considered as established, that ulceration of the small intestines does not take place in the acute form of typhus; but that the diarrhœa which happens as a sequel to it depends upon ulceration, hypertrophy, or inflammation of the ileum, cœcum, or colon. *Peyer's glands are usually unaffected in any form of ship fever.*"

The treatment pursued by the author seems to have been a highly judicious one. We need hardly cite it, as it contains nothing particularly new to practitioners. His opinion in reference to the propriety of venesection, is, that in uncomplicated cases it is not advisable, and if we understand him rightly, he seems never to have taken away blood, except by leeches or cups for the purpose of combating a severe pneumonia or bronchitis. The chief reliance was placed upon the administration of stimulants, the proper quantity being determined by the effect upon the pulse. The diet during convalescence was regulated with the greatest care, as a slight indiscretion was very frequently the cause of a relapse or brought on the fatal diarrhœa. When the weather permitted, the patients were allowed to walk into the open air as soon as they were able, and improved, in consequence, much more rapidly than when confined.

We must refer our readers to the original dissertation for the proof of the conclusions at which the author arrives, viz:

"Ship fever is identical with the true typhus of Great Britain. It is not identical with the typhoid fevers of France and New England, but an entirely distinct disease.

It is most fatal in its secondary forms.

And lastly, that its contagious properties may be greatly controlled, if not destroyed, by suitable sanitary measures." M. S.

